# USVSEG: A robust method for segmentation of ultrasonic vocalizations in rodents

**DOI:** 10.1371/journal.pone.0228907

**Published:** 2020-02-10

**Authors:** Ryosuke O. Tachibana, Kouta Kanno, Shota Okabe, Kohta I. Kobayasi, Kazuo Okanoya

**Affiliations:** 1 Department of Life Sciences, Graduate School of Arts & Sciences, The University of Tokyo, Tokyo, Japan; 2 Laboratory of Neuroscience, Course of Psychology, Department of Humanities, Faculty of Law, Economics and the Humanities, Kagoshima University, Kagoshima, Japan; 3 Division of Brain and Neurophysiology, Department of Physiology, Jichi Medical University, Tochigi, Japan; 4 Graduate School of Life and Medical Sciences, Doshisha University, Kyoto, Japan; Université de Bordeaux and Centre National de la Recherche Scientifique, FRANCE

## Abstract

Rodents’ ultrasonic vocalizations (USVs) provide useful information for assessing their social behaviors. Despite previous efforts in classifying subcategories of time-frequency patterns of USV syllables to study their functional relevance, methods for detecting vocal elements from continuously recorded data have remained sub-optimal. Here, we propose a novel procedure for detecting USV segments in continuous sound data containing background noise recorded during the observation of social behavior. The proposed procedure utilizes a stable version of the sound spectrogram and additional signal processing for better separation of vocal signals by reducing the variation of the background noise. Our procedure also provides precise time tracking of spectral peaks within each syllable. We demonstrated that this procedure can be applied to a variety of USVs obtained from several rodent species. Performance tests showed this method had greater accuracy in detecting USV syllables than conventional detection methods.

## Introduction

Various species in the rodent superfamily *Muroidae* (which includes mice, rats, and gerbils) have been reported to vocalize ultrasonic sounds in a wide range of frequencies up to around 100 kHz [1]. Such ultrasonic vocalizations (USVs) are thought to be associated with specific social behaviors. For several decades, laboratory mice (*Mus musculus domesticus* and *Mus musculus musculus*) have been reported to produce USVs as part of courtship behaviors [2,3]. Their vocalizations are known to form a sequential structure [4] which consists of various sound elements, or ‘syllables’. Almost all USV syllables in mice exhibit spectral peaks between 50–90 kHz with a time duration of 10–40 ms, though slight differences in the syllable spectrotemporal pattern were observed among different strains [5]. On the other hand, it has been also well described that laboratory rats (*Rattus norvegicus domesticus*) produce USV syllables which have two predominant categories: one has a relatively higher frequency (around 50 kHz) with short duration (a few tens of milliseconds), and the other has a lower frequency (~22 kHz) but much a longer duration. These two USV syllables are here named as ‘pleasant’ and ‘distress’ syllables since these are generally considered to be indicators of positive and negative emotional states, respectively [6–9]. This categorization appears to be preserved in different strains of rats, though a slight difference in duration has been reported [10]. In another rodent family, Mongolian gerbils (*Meriones unguiculatus*), vocalizations have also been extensively studied as animal models for both the audio-vocal system and for social communication [11–14]. They produce various types of USV syllables with a frequency range up to ~50 kHz and distinct spectrotemporal patterns [15,16].

In general, rodent USVs have been thought to have ecological functions for male-to-female sexual display [2,3,17–20], emotional signal transmission [21–26], and mother-infant interactions [27–30]. Mouse USVs can be discriminated into several subcategories according to their spectrotemporal patterns [31–35], and these patterns could predict mating success [35,36], though subcategories are not consistent between studies. Their USV patterns are innately acquired rather than a learned behavior [34,37], though sociosexual experience can slightly enhance the vocalization rate [38]. In rat USVs, the pleasant (~50 kHz) and distress (~22 kHz) calls have been suggested to have a communicative function since these calls can transmit the emotional states of the vocalizer to the listener and can modify the listener’s behavior such as mating [26,39], approaching [40], or defensive behavior [41,42]. It has been suggested that perception of these calls can also modulate the listeners’ affective state [21]. Further discrimination of subcategories within the pleasant call has been studied to better understand their functional differences in different situations [6–9].

From these characteristics and functions, rodent USVs are expected to provide a good window for studying sociality and communication in animals. Mouse USVs have been used for studying disorders of social behavior, with a particular focus on autism spectrum disorder [43–46]. Thanks to recent genetic manipulation techniques, social disorders can be modeled in mice and then studied directly through USV analysis to quantify social behavior. On the other hand, studies utilizing rat USVs have focused on elucidating the neural mechanisms for the emotional system [6–9], maternal behavior [47–49], and social interactions [50]. USVs of other species in the same superfamily of rodents have also been studied as a variety of research models, including parental behaviors, auditory perception and vocal motor control in gerbils [11,51,52]. Thus, a unified analysis tool for analyzing rodent USVs is helpful to transfer knowledge obtained across different species.

Previous studies have proposed analysis toolkits such as VoICE [53], MUPET [54] or DeepSqueak [55], which are successful when the recorded sounds have a sufficiently high signal-to-noise ratio. These analysis tools can be less effective when recordings are contaminated with background noise introduced during recording. Noise can be short and transient (e.g., scratching sounds) or stationary (e.g., noise produced by fans or air compressors). Such noise greatly deteriorates the segmentation of USV syllables, and smears acoustical features (e.g., peak frequency) of the segmented syllables, possibly reducing the reliability of classification of vocal categories and quantification of acoustical features of vocalizations.

**Fig 1:**
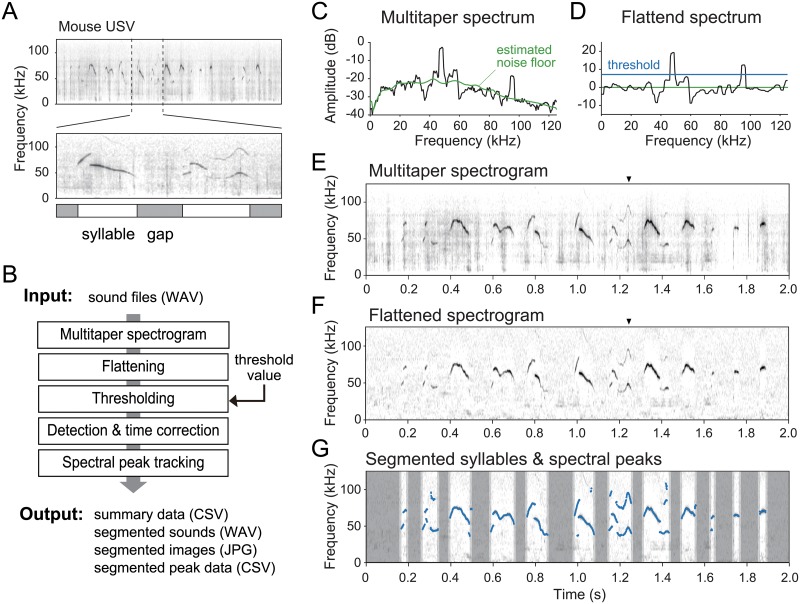
Spectrogram of a rodent ultrasonic vocalization (USV) and proposed method for detection of vocal elements in continuously recorded data. (A) Example spectrogram of a mouse vocalization. The brief segment of vocalization (a few ten to a few hundred milliseconds) is defined as a ‘syllable’, and the time interval between two syllables is called a ‘gap’. (B) Schematic diagram of the proposed signal processing procedure. (C, D) Example of a multitaper spectrum and a flattened spectrum, respectively. The flattening process subtracts an estimated noise floor (green line), and the segmentation process detects spectral components above the defined threshold (blue line) as syllables. Example multitaper (E) and flattened (F) spectrograms obtained from a recording of a male mouse performing courtship vocalizations to a female mouse. (G) Processed result of USV data (same as E,F) showing detected syllable periods and spectral peak traces of the syllables (blue highlighted dots). Dark gray zones show non-syllable periods and light gray zones indicate a margin inserted before and after a syllable period.

Despite a variety of behaviors and functions among species, rodent USVs generally tend to exhibit a single salient peak in the spectrum, with few weak harmonic components, if any (see Fig 1A for example). This tendency is associated with a whistle-like sound production mechanism [56]. From a sound analysis point of view, this characteristic provides a simple rule for isolating USV sounds from background noise; that is, narrow-band spectral peaks can be categorized as vocalized sounds whereas broadband spectral components can be categorized as the background. Thus, emphasizing the spectral peaks while flattening the noise floor should improve discrimination of vocalized signals from background noise.

Here, we propose a signal processing procedure for robust detection of USV syllables in recorded sound data by reducing acoustic interference from background noise. Additionally, this procedure is able to track multiple spectral peaks of the segmented syllables. This procedure consists of five steps (see Fig 1B): (i) make a stable spectrogram via the multitaper method, which reduces interaction of sidelobes between signal and stochastic background noise, (ii) flatten the spectrogram by liftering in the cepstral domain, which eliminates both pulse-like transient noise and constant background noise, (iii) perform thresholding, (iv) estimate onset/offset boundaries, and (v) track spectral peaks of segmented syllables on the flattened spectrogram. The proposed procedure is implemented in a GUI-based software (“USVSEG”, implemented as MATLAB scripts; available from https://sites.google.com/view/rtachi/resources), and it outputs segmented sound files, image files, and spectral peak feature data after receiving original sound files. These output files can be used for further analyses, for example, clustering, classification, or behavioral assessment by using other toolkits.

The present study demonstrated that the proposed procedure can be successfully applied to a variety of USV syllables produced by a wide range of rodent species (see Table 1). It achieves nearly perfect performance for segmenting syllables in a mouse USV dataset. Further, we confirmed that our procedure was more accurate in segmenting USVs, and more robust against elevated background noise than conventional methods.

**Table 1:** Rodent USV dataset for performance tests.

Species	Strain and Condition	Data ID	File duration (s)	# syllables
Mouse (Mus musculus)	C57BL/6J male courtship	A / Aco59_2	113.4	333
		B / Aco59_2	115.5	85
		C / Can15-1	97.6	371
		D / Can15-1	97.0	217
		E / Can16-1	135.9	394
		F / Can16-1	118.7	171
		G / Can9_2	104.7	420
		H / Can9_2	205.9	306
		I / Aco65-1	98.6	383
		L / Can3_1	104.9	119
	BALB/c male courtship	BALB128-4	60.0	168
		ClnBALB124-4	53.0	203
	Shank2- male courtship	Shank2_S2-4-65	65.1	261
		Shank2_S2-4-103	65.1	193
		Shank2_S2-4-108	65.1	326
	C57BL/6J isolated pup call	Rin3-1pup	175.0	62
		Rin3-3pup	175.0	41
		Rin3-7pup	175.0	117
		Rin3-8pup	175.0	112
		Rin3-9pup	175.0	74
Rat (*Rattus norvegicus domesticus*)	LEW/CrlCrlj pleasant call	a14	33.2	100
		a18	51.6	100
		a42	28.7	100
	LEW/CrlCrlj distress call	a55	110.0	37
		a57	142.0	57
		a58	100.0	29
		a60	100.0	32
Gerbil (Meriones unguiculatus)	male courtship	uFMdata	27.5	124
		uSFMdata	59.0	112

## Results

### Overview of USV segmentation

Rodents USVs consist of a series of brief vocal elements, or ‘syllables’ (Fig 1A), in a variety of frequency ranges, depending on the species and situation. For instance, almost all mouse strains vocalize in a wide frequency range of 20–100 Hz, while rat USVs show a focused frequency around 20–30 kHz when they are in distress. These vocalizations are sometimes difficult to detect visually in a spectrogram because of unavoidable background noise. Such situations provide a challenge to the detection and segmentation of each USV syllable from recorded sound data. Here, we assessed a novel procedure consisting of several signal processing methods for segmentation of USV syllables (Fig 1B). A smooth spectrogram of recorded sound was obtained using the multitaper method (Fig 1C and 1E) and was flattened by cepstral filtering and median subtraction (Fig 1D and 1F). The flattened spectrogram was binarized with a threshold that was determined in relation to the estimated background noise level. Finally, the signals that exceeded the threshold were used to determine the vocalization period (Fig 1G). Additionally, our procedure detects spectral peaks at every timestep within the segmented syllable periods. In this procedure, users only need to adjust the threshold value based on the signal-to-noise ratio of the recording, and they do not need to adjust any other parameters (e.g., maximum and minimum limits of syllable duration and frequency) once appropriate values for individual animals have been determined. Note that we provided heuristically determined parameter sets as reference values (see Table 2).

**Table 2:** Chosen parameter sets for different species and situations.

Species/Strain/Condition	min. frequency (kHz)	max. frequency (kHz)	min. duration (ms)	max. duration (ms)	min. gap (ms)	threshold value (σ)
Mice (C57BL/6J)	40	120	3	300	30	4.0
Mice (BALB/c)	40	120	3	300	30	3.5
Mice (Shank2-)	40	120	3	300	30	3.5
Mice (C57BL/6J pup)	40	120	3	300	30	3.0
Rats (pleasant call)	20	100	3	500	40	3.0
Rats (distress call)	12	40	100	3000	40	6.0
Gerbils	20	60	5	300	30	4.0

### Searching for an optimal threshold

**Fig 2:**
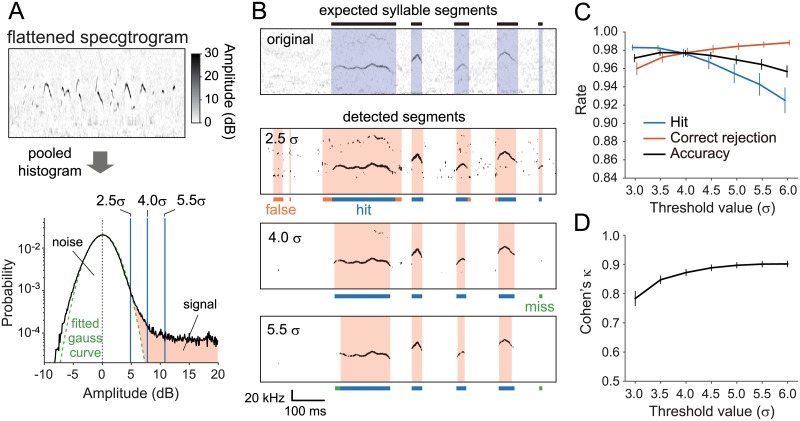
Relationship between thresholding and segmentation performance for mouse USVs. (A) Computation scheme of the threshold value. All data points (pixels) of the flattened spectrogram were pooled and used to make a histogram as a function of amplitude in dB (black line). The background noise distribution was parameterized by a standard deviation (σ) of the gaussian curve (green broken line). The threshold value was defined as a weighting factor of σ (shown as blue vertical lines for example values: 2.5, 4.0 and 5.5). (B) Example segmentation results for threshold values at 2.5, 4.0 and 5.5 (σ). Uppermost panel shows the original flattened spectrogram with segmented periods as syllables performed by a human expert (blue shaded area). Lower three panels depict thresholded binary images and results of our automatic segmentation for three different threshold values (orange shaded areas). Typically, the false detection rate decreases and the miss rate increases as the threshold value increases. (C) Segmentation performance of our procedure as a function of threshold value. The hit rate (blue line) tends to decrease, while the correct rejection (red) tends to increase as the threshold value increases. The accuracy score (black) showed a balanced index of the performance. (D) Cohen’s κ index as a function of threshold value, showing consistency of detections between the automatic and the manual segmentations. The κ index tends to increase as the threshold value increases.

To assess the relationship between the threshold parameter and segmentation performance, we validated the segmentation performance of our procedure on a mouse USV dataset (Fig 2). The actual threshold was defined as the multiplication of a weighting factor (or “threshold value”) and the background noise level, which was quantified as the standard deviation (σ) of an amplitude histogram of the flattened spectrogram (Fig 2A; see Methods for details). With low or high threshold values, the segmentation procedure could miss weak vocalizations, or mistakenly detect noise as syllables, respectively (Fig 2B). To find an optimal threshold value for normal recording conditions, we conducted a performance test on a dataset including 10 recorded sound files obtained from 7 mice that had onset/offset timing information defined manually by a human expert (Table 1). We calculated hit and correct rejection (CR) rates to quantify and compare the consistency of segmentation by the proposed procedure with that of manual processing (see Methods). The results showed a tendency for the hit rate to decrease and the correct rejection rate to increase as the threshold value increased (Fig 2C). We also quantified the consistency by an inter-rater consistency index, Cohen’s *κ* [57]. As the result, we found the *κ* index tended to increase along increasing threshold values, and was sufficiently high (> 0.8) [58] when the threshold value was 3.5 or more (Fig 2D). Note that we used an identical parameter set for the performance tests in this (see Table 2) with varying the threshold value only.

### Segmentation performance for various USVs

**Fig 3:**
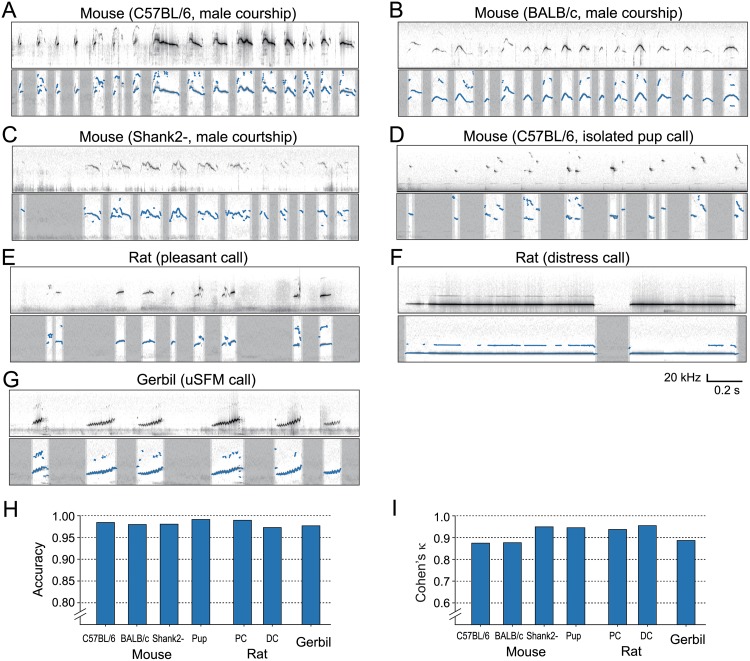
Example results of segmentation and spectral peak tracking on various rodent species. (A-C)Mice courtship calls obtained from three different strains: (A) C57BL/6, (B) BALB/c, (C) a disease model for mutation in ProSAP1/Shank2 proteins (“Shank2-”). (D) Pup calls obtained an isolated juvenile mouse (C57BL/6). (E-F) Rats calls in the context of pleasant (E) and distress (F) situations. (G) Representative USV sounds of gerbils, named upward sinusoidal frequency modulated (uSFM) calls. (H-I) Detection performance on all seven dataset conditions. All conditions showed more than 0.95 accuracy scores (H) and sufficiently high κ-index values (I) on the dataset (see Methods for details).

To demonstrate applicability of the proposed procedure for a wide variety of rodent USVs, we tested segmentation performance on the USVs of two strains of laboratory mice (C57BL/6J and BALB/c), two different call types from laboratory rats (PC and DC), and USV syllables of gerbils, respectively. We conducted performance tests for each dataset using manually detected onset/offset information (Table 1). Note that PC and DC in rats show a remarkable difference in both duration and frequency range even when produced in the same animal; thus we treated the two calls independently and used different parameter sets for them. Similar to threshold optimization, we calculated hit and CR rates to quantify matching of segmentation between automatic methods and manual segmentation by human experts. Results show that when using heuristically chosen parameter sets (Table 2), our procedure has over 0.95 accuracy in segmenting various USV syllables (Fig 3). Slight variability in the accuracy and *κ* index was observed across conditions, and this can be explained by differences in the background noise level during recording (as shown in the spectrogram for gerbil vocalizations containing scratch noises).

### Comparison with conventional methods

**Fig 4:**
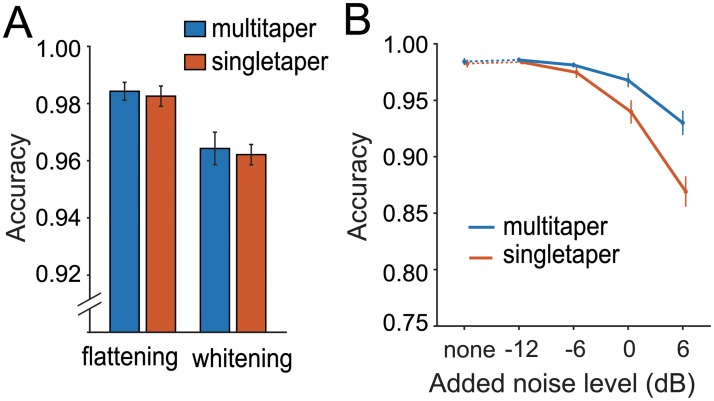
Segmentation performance of various combinations of signal processing methods. (A) Accuracy scores of segmentation on flattened or whitened spectrogram produced by a multitaper (blue) or singletaper (red) method. We used the hanning window as the single taper condition. (B) Performance sensitivity to additive noise. Segmentation with multitaper (blue) and single-taper (red) spectrograms against experimentally added background noise. White noise was added to the original data at levels of −12, −6, 0, or 6 dB before processing.

We compared our procedure with conventional signal processing methods (Fig 4), which include a single-window (or “singletaper”) method for generating the spectrogram, and long-term spectral subtraction (“whitening”) and have been previously used in other segmentation procedures [59]. As in this previous study, we used the hanning window as a typical singletaper to generate the spectrogram. The performance test was carried out with four conditions consisting of combinations of two windows (multitaper vs. singletaper) and two noise reduction methods (flattening vs whitening) (see “Comparison with conventional methods” in Methods). The dataset used for searching for the optimal threshold was also used for this performance test. Results demonstrated greater performance with flattening than whitening, and slightly higher performance in multitaper compared to singletaper spectrograms (Fig 4A). A statistical test showed a significant effect of noise reduction method (two-way ANOVA; F(1,36) = 19.02, p < 0.001), but not of windowing method (F(1,36) = 0.17, p = 0.681), and there was no significant interaction between them (F(1,36) = 0.00, p = 0.960). Further, to determine robustness of the segmentation methods against noise, we added white noise to the sound dataset at levels of −12, −6, 0, and 6 dB higher than the original sound (see “Noise addition” in Methods), and ran the performance test again. In particular, we compared performance between the multitaper and singletaper spectrograms using only flattening for the noise reduction. The result of this test clearly showed that the multitaper method was more robust for degraded signal-to-noise situations than the singletaper method (Fig 4B). The statistical test showed significant main effects of both additive noise level and windowing method with significant interaction between them (two-way ANOVA; noise level: F(4,90) = 51.54, p < 0.001; window: F(1,90) = 19.59, p < 0.001; interaction: F(4,90) = 6.42, p < 0.001).

## Discussion

The proposed procedure showed nearly perfect segmentation performance for variable USV syllables of a variety of species and strains in the rodent superfamily. The procedure was designed to emphasize vocal components in the spectral domain while reducing variability of background noise, which inevitably occurs during observation of social behavior, and usually interferes with the segmentation process. This process helped to discriminate vocal signals from the background by thresholding according to the signal-to-noise ratio. Additionally, our procedure also provides a precise tracking of spectral peaks within each vocalized sound. The proposed method was more robust than the conventional method for syllable detection, in particular, under elevated background noise levels. These results demonstrated that this procedure can be generally applied to segment USVs of several rodent species.

Our procedure was designed to emphasize distribution differences between vocal signals and background noise, under the assumption that rodent USV signals generally tend to have narrow-band sharp spectral peaks. In this process, we employed the multitaper method which uses multiple windows for performing spectral analysis (Thomson 1982), and has been used in vocal sound analyses for other species, e.g., songbirds [56]. We also introduced the spectral flattening process in which the broadband spectrum in each timestep was flattened by cepstral filtering. As we demonstrated in the performance comparison tests, a combination of multitaper windowing with flattening showed better performance than the conventional method of single-taper windowing with long-term spectral subtraction that has been used in a previous mouse USV analysis [59]. In particular, the difference in performance was seen under degraded signal-to-noise conditions. Note that our experimental results did not focus on applicability for lower-frequency (i.e. <20 kHz), harmonic-rich, or harsh noise-like vocalizations since these types of sounds are outside of the scope of the processing algorithm.

We here employed a redundant way to represent the spectral features of USVs, and exporting up to 3 candidates for spectral peaks for every timestep. This provides additional information about harmonics, as well as an appropriate way to treat “jumps,” which are sudden changes in the spectral peak tracks [4]. Researchers have attempted to distinguish syllables into several subcategories according to their spectrotemporal features so they can analyze sequential patterns to understand their syntax [32,35,36,60]. The procedure proposed in the present study will allow for better categorization of USV syllable subtypes.

## Methods

### Proposed procedure

Our procedure consists of five steps: multitaper spectrogram generation, flattening, thresholding, detecting syllable onset/offset, and spectral peak tracking. In particular, the multitaper method and flattening were core processes for suppressing the variability of background noise as described below in detail.

#### Multitaper spectrogram generation

We used the multitaper method [61] for obtaining the spectrogram to improve signal salience against background noise distribution. Multiple time windows (or tapers) were designed as a set of 6 series of discrete prolate spheroidal sequences with the time half bandwidth parameter set to 3 [62]. The length of these windows was set to 512 samples (~2 ms for 250 kHz sampling rate). In each time step, the original waveform was multiplied by all six windows and transformed into the frequency domain. The six derived spectrograms were averaged into one to obtain a stable spectrotemporal representation. This multitaper method reduces background noise compared to a typical single-taper spectrogram, while widening the bandwidth of signal spectral peaks.

#### Flattening

To emphasize spectral peaks for detectability of vocalization events, we reduced the variability of background noise by flattening the spectrogram. This flattening consists of two processes. First, transient broadband (or impulse-like) noises were reduced by liftering in every time step, in which gradual fluctuation in the frequency domain, or spectral envelope was filtered out by replacing the first three cepstral coefficients with zero. This process can emphasize spectral peaks of rodent USVs since they are very narrow-band and have few or no harmonics. Then, we calculated a grand median spectrum that had median values of each frequency channel, and subtracted it from the liftered spectrogram.

#### Thresholding and detection

After flattening, we binarized the flattened spectrogram image at a threshold which was determined based on the estimated background noise level. The threshold was calculated as the multiplication of a weighting factor (or “threshold value”) and the standard deviation (σ) of a background distribution (Fig 2A). The σ value was estimated from a pooled amplitude histogram of the flattened spectrogram as described in a previous study for determining onsets and offsets of birdsong syllables [63]. The threshold value is normally chosen from 3.5–5.5 and can be manually adjusted depending on the background noise level. After binarization, we counted the maximum number of successive pixels along the frequency axis whose amplitude exceeded the threshold in each time frame, and considered the time frame to include vocalized sounds when the maximum number counted was 5 or more (corresponding to a half bandwidth of the multitaper window).

#### Timing correction

A pair of detected elements split by a silent period (or gap) with a duration less than the predefined lower limit (“gap min”) was integrated to omit unwanted segmentation within syllables. We usually set this lower limit for a gap around 3–30 ms according to specific animal species or strains. Then, sound elements with a duration of more than the lower limit (“dur min”) were judged as syllables. If the duration of an element exceeded the upper limit (“dur max”), then the element was excluded. These two parameters (dur min and max) were differentially determined for different species, strains, and situations. The heuristically determined values of these parameters are shown in a table (Table 2) for reference.

#### Spectral peak tracking

We also implemented an algorithm for tracking multiple spectral peaks as an additional analysis after segmentation. Although the focus of our study is temporal segmentation of syllables, we briefly explain this algorithm as follows. First, we calculated the degree of salience of spectral peaks by convolving a second-order differential spectrum of the multitaper window itself into the flattened spectrum along the frequency axis. This process emphasizes the steepness of spectral peaks in each time frame. Then, the strongest four local maxima of spectral saliency were detected as candidates. We grouped the four peak candidates to form a continuous spectral object according to their time-frequency continuity (within 5% frequency change per time frame). If the length of the grouped object was less than 10 time points, the spectral peak data in the object was excluded as a candidate for a vocalized sound. At the final step, the algorithm outputs up to three peaks in each time step.

#### Output files

The software can output a variety of processed data in multiple forms (Fig 1B). Segmented syllables are saved as sound files (WAV format), and image files of either flattened or original spectrograms (JPG format). A summary file (CSV) contains onset and offset time points and an additional three acoustical features for each segmented syllable: duration, max-frequency (maxfreq), and max-amplitude (maxamp). Maxfreq is defined as the peak frequency of the time frame that has the highest amplitude in that syllable, and maxamp is the amplitude value of the maxfreq. These features are widely used in the field of USV studies [35–37]. Furthermore, the software generates the peak frequency trace of each syllable so that users can perform post-processing after segmentation to obtain additional features.

### Dataset

For testing the segmentation performance of our procedure, we prepared datasets consisting of recorded sounds and manually detected onset/offset timing of syllables for three species in the rodent superfamily (Table 1). The manual segmentation for each species was performed by a different human expert. These experts segmented sound materials by visual inspection of a spectrogram, independently of any automatic segmentation system. They were not informed about any of the results of our procedure beforehand. Finally, we collected segmented data for each condition (species, strains, or contexts) as described below. For all species/strain/context conditions, ultrasonic sounds were recorded using a commercial condenser microphone and an A/D converter (Ultra-SoundGate, Avisoft Bioacoustics, Berlin, Germany; SpectoLibellus2D, Katou Acoustics Consultant Office, Kanagawa, Japan). All data were resampled at 250 kHz to have the same sampling rates before starting performance tests for consistency across datasets, though our procedure can be applied to data with much higher sampling rates. The whole dataset is available online (https://doi.org/10.5281/zenodo.3428024).

#### Mice

We obtained 10 recording sessions of courtship vocalizations from 6 mice (*Mus musculus*; C57BL/6J, adult males), under the same condition and recording environment as described in our previous work [38]. Briefly, the microphone was set 16 cm above the floor with a sampling rate of 400 kHz. Latency to the first call was measured after introducing an adult female of the same strain into the cage and then ultrasonic recording was performed for one additional minute. The data recorded during the first minute after the first ultrasound call was analyzed for the number of calls. For all recording tests, the bedding and cages for the males were changed one week before the recording tests, and these home-cage conditions were maintained until the tests were completed. These sound data files were originally recorded as part of other experiments (in preparation), and shared on mouseTube [64] and Koseisouhatsu Data Sharing Platform [65]. Note that we chose 10 files from the shared data and excluded two files (“J” and “K”) which did not contain enough syllables for the present study. To assess the applicability of our procedure for a wider range of strains and situations, we also obtained data from another strain (“BALB/c”), a disease model (“Shank2-”), and an isolated juvenile’s pup call (“Pup”). For the disease model, we used the dataset of ProSAP1/Shank2-/- mice [33], which was also available on mouseTube. These mice have mutated ProSAP1/Shank2, which is one of the synaptic scaffolding proteins mutated in patients with autism spectrum disorders (ASD). The experimental procedure used for BALB/c mice was the same as for C57BL/6J mice. For Shank2- mice, the procedure was similar but see reference for details [33].

#### Mouse pups

For recording pup USVs, we used C57BL/6J mice at postnatal day 5–6. The microphone was set 16 cm above the floor with a sampling rate of 384 kHz. After introducing a pup into a clean cage from their nest, ultrasonic recording was performed for three minutes. These sound data files were originally recorded as part of other experiments (in preparation).

#### Rats

The pleasant call (PC) or distress call (DC) was recorded from an adult female rat (*Rattus norvegicus domesticus*; LEW/CrlCrlj, Charles River Laboratories Japan). For the recording of PC, the animal was stroked by hand on the experimenter’s lap for around 5 minutes. To elicit DC, a different animal was transferred to a wire-topped experimental cage and habituated to the cage for 5 minutes. Then, the animal received air-puff stimuli (0.3 MPa) with an inter-stimulus interval of 2 s to the nape from a distance of approximately 5–10 cm. Immediately after 30 air-puff stimuli were delivered, USVs were recorded for 5 min. These vocalizations were detected by a microphone placed at a distance of approximately 15–20 cm from the target animal. The detected sound was digitally recorded at a sampling rate of 384 kHz.

#### Gerbils

Vocalizations of the Mongolian gerbil (*Meriones unguiculatus*) were recorded via a microphone positioned 35 cm above an animal cage that was positioned in the center of a soundproof room. The sound was digitized at a sampling rate of 250 kHz. This sound data was originally obtained as part of a previous study [15]. Here, we targeted only calls with fundamental frequencies in the ultrasonic range (20 kHz or more; i.e., upward FMs and upward sinusoidal FMs), which are often observed under conditions that appear to be mating and non-conflict contexts [15].

### Performance tests

#### Segmentation performance score

We quantified segmentation performance of our software by calculating accuracy scores. First, onset and offset timestamps of detected syllables for each data file were converted into a boxcar function which indicated syllable detection status by 1 (detected) and 0 (rejected) in every 1-ms time step. We counted the number of time frames which contained true-positive or true-negative detections as hit and correct-rejection counts, respectively. Then, the accuracy score was calculated as an average of hit and correct-rejection rates. We additionally calculated an inter-rater agreement score, Cohen’s κ [58], to assess degrees of agreement between our software and human experts by the following formula: (*p*_a_−*p*_c_) / (1 –*p*_c_), where *p*_a_ indicates the accuracy, and *p*_c_ shows an expected probability to coincide two raters by chance.

#### Threshold optimization

When the threshold was set too high, the segmentation procedure would miss weak vocal sounds, or mistakenly detect noises as syllables. To find an optimal threshold value for normal recording conditions, we assessed segmentation performance on a dataset for mice USVs by changing the threshold value. For this test, we used 10 files of C57BL/6J mice from the dataset. We varied the threshold value from 3.0 to 6.0 with 0.5 steps. The optimal value was defined as the value which showed a peak in the accuracy index.

#### Comparison with conventional methods

To determine to what extent our procedure improved the detection performance from conventional methods, we compared the performance of four conditions in which two processing steps were swapped with conventional ones. For conventional methods, we employed a normal windowing method (“singletaper” condition) using the hanning window to replace the multitaper method for making the spectrogram. We also used long-term spectral subtraction (“whitening” condition) to replace the flattening process. These two methods have been used as standard processing methods for signal detection algorithms [59]. Here, we swapped one or both methods (windowing and noise reduction) between ours and conventional ones to make four conditions: multitaper+flattening, multitaper+whitening, singletaper+flattening, and singletaper+whitening. Then, the performance of each condition was tested on the mouse USV dataset that was used for the threshold optimization test. Note that the threshold for bandwidth in the detecting process after the binarization was adjusted to 3 for the singletaper method (it was 5 for the multitaper) to correspond to its half bandwidth. A two-way repeated measures ANOVA was performed on the windowing factor (singletaper vs multitaper) and the noise-reduction factor (flattening vs whitening) with a significance threshold *α* = 0.05.

#### Noise addition

As an additional analysis for assessing the robustness against noise, we carried out the performance test again on the multitaper+flattening and singletaper+flattening conditions by adding white noise to the original sound data at levels of −12, −6, 0, and 6 dB, referring to the root-mean-square of the original sound amplitude. We have not tested the whitening method here since the method showed clearly lower performance than the flattening method in the original performance test. A two-way repeated measures ANOVA was performed on the windowing factor (singletaper vs multitaper) and the noise-level factor (none, −12, −6, 0, 6 dB) with a significance threshold *α* = 0.05.

### Ethical information

All procedures for recording vocalizations were approved by the Ethics Committee of Azabu University (#130226–04) or Kagoshima University (L18005) for mice, and the Animal Experiment Committee of Jichi Medical University (#17163–02) for rats.
